# Intravenous Hydroxocobalamin for Cyanide Poisoning From Smoke Inhalation: A Comprehensive Scoping Review

**DOI:** 10.1016/j.acepjo.2026.100340

**Published:** 2026-02-27

**Authors:** Robert Dunne, Jeffrey M. Goodloe, James J. Augustine, Tommy Begres, Remle P. Crowe, Erica Carney

**Affiliations:** 1 St. John Hospital and Medical Center and Wayne State University School of Medicine, Detroit, Michigan, USA; 2 University of Oklahoma School of Community Medicine, Tulsa, Oklahoma, USA; 3 Wright State University Boonshoft School of Medicine, Dayton, Ohio, USA; 4 SERB Pharmaceuticals, West Conshohocken, Pennsylvania, USA; 5 ESO, Austin, Texas, USA; 6 Truman Medical Center and University of Missouri-Kansas City School of Medicine, Kansas City, Missouri, USA

**Keywords:** cyanide poisoning, hydroxocobalamin, inhalation injury, smoke inhalation

## Abstract

Smoke inhalation from closed-space fire can result in hydrogen cyanide (HCN) exposure, an underrecognized contributor to morbidity and mortality in burn and smoke inhalation victims. Hydroxocobalamin, a vitamin B12 precursor, is recommended by the American Heart Association for suspected HCN poisoning; however, evidence regarding its real-world use, particularly in the prehospital setting, remains fragmented. This scoping review aims to identify the existing literature on hydroxocobalamin administration for HCN toxicity secondary to smoke inhalation. BIOSIS Previews, Embase, PubMed, and MEDLINE were searched from inception through January 1, 2025. Data extracted included patient demographic characteristics, hydroxocobalamin dosing, pretreatment and posttreatment clinical data, and adverse events (AEs). We reported descriptive statistics. Of 591 titles and abstracts screened for eligibility, 34 full-text papers were reviewed, and 21 papers describing 512 unique patients were included. Hydroxocobalamin was administered in the prehospital setting in 235 (45.9%) of patients. Most patients received either 5 or 10 g total doses. Pretreatment HCN and blood lactate levels were described in 6 and 12 studies, respectively. Expected AEs included chromaturia and skin discoloration; acute kidney injury was also reported. Among 482 patients with known survival status, 318 (66.0%) survived to hospital discharge. Collectively, these results suggest that hydroxocobalamin was generally well-tolerated and associated with favorable patient outcomes, but evidence remains observational and heterogeneous. This review underscores critical gaps and highlights variability in the real-world use of hydroxocobalamin for HCN toxicity. Additional work is needed to further understand and address barriers to hydroxocobalamin use.

## 1 Introduction

### 1.1 Background

In the United States (US), fires and burns remain the fifth leading cause of unintentional injury-related deaths.^1^ Although the number of residential fires declined from 2022 to 2023, civilian injuries and deaths from home fires increased.^2^^,^^3^ Many of these fatalities may be attributable not only to thermal injury but also to inhalation of products of combustion (smoke and toxic gases), including hydrogen cyanide (HCN).^1^^,^^4^ HCN toxicity is often underrecognized, and clinical management remains variable in the prehospital and emergency department settings.

### 1.2 Importance

Modern building materials, furnishings, and textiles, rich in nitrogen-containing synthetic compounds, generate HCN and other toxic gases when burned.^4^, ^5^, ^6^ In closed-space fires, this greatly amplifies the risk of HCN exposure for both victims and rescuers.^7^^,^^8^ When HCN overwhelms the body’s natural detoxification pathways, cellular respiration halts, leading to cardiovascular collapse and death within minutes if untreated.^9^, ^10^, ^11^

Initial prehospital management of suspected HCN toxicity from smoke inhalation focuses on removal from the exposure source, cardiopulmonary support, provision of 100% supplemental oxygen, and administration of timely antidotal therapy to improve the chance of survival.^9^^,^^12^, ^13^, ^14^, ^15^, ^16^ Historically available antidotes (amyl nitrite, sodium nitrite, and sodium thiosulfate) carry significant adverse effects.^13^

Hydroxocobalamin, a vitamin B12 precursor administered intravenously, represents a major advancement in the treatment of suspected HCN poisoning. This cobalt-containing compound binds HCN to form cyanocobalamin, which is excreted in urine.^15^^,^^17^^,^^18^ Unlike older antidotes, hydroxocobalamin does not induce methemoglobinemia and thus preserves oxygen-carrying capacity, even in patients with concomitant carbon monoxide (CO) poisoning.^17^ Because of its safety profile, hydroxocobalamin is the preferred antidote for prehospital administration and is recommended by the American Heart Association (AHA) poisoning guidelines for patients with suspected HCN poisoning.^6^

Despite US Food and Drug Administration (US FDA) approval in 2006 and endorsement in national guidelines, hydroxocobalamin use in the US remains inconsistent.^19^ Variability in access, protocol adoption, and clinician familiarity may delay treatment. However, the existing evidence describing hydroxocobalamin use, particularly in the prehospital setting, is fragmented and lacks synthesis across studies. This knowledge gap limits understanding of how, when, and in whom hydroxocobalamin is being used, as well as its safety and outcomes in real-world treatment of fire-related cyanide toxicity.

### 1.3 Goals of This Investigation

The purpose of this scoping review was to systematically map and synthesize the published evidence on hydroxocobalamin for HCN toxicity due to smoke inhalation. Specifically, we sought to describe patient populations, dosing practices, timing and setting of administration (prehospital versus hospital settings), and reported outcomes and adverse events (AEs). Additionally, we aimed to identify evidence gaps and practical barriers that may hinder optimal use of hydroxocobalamin in prehospital and hospital care. Understanding these gaps and practical barriers may inform future research, policy, and system-level efforts to improve timely recognition and treatment of HCN toxicity.

## 2 Methods

This scoping review was conducted in accordance with the Preferred Reporting Items for Systematic Reviews and Meta-Analyses extension for Scoping Reviews (PRISMA-ScR) guidelines.

### 2.1 Search Strategy

A comprehensive search of BioSciences Information Service (BIOSIS) Previews, Embase, PubMed, and MEDLINE was performed from inception up to January 1, 2025, without restriction by language. Search terms combined key concepts related to cyanide exposure and hydroxocobalamin treatment, including (“cyanide” or “hydroxocobalamin”) AND (“inhalation injury” or “smoke inhalation”). Reference lists of included studies were manually screened to identify additional citations. Gray literature, including nonpeer-reviewed sources, was also reviewed. Articles from authors’ institutional or personal reference files were considered, and translations of non-English articles were obtained.

### 2.2 Inclusion and Exclusion Criteria

Eligible studies included clinical trials, observational analyses, open-label studies, and case reports/series that reported on the use of hydroxocobalamin for the management of HCN poisoning associated with inhalation injury in adult patients. Studies were excluded if they (1) evaluated hydroxocobalamin use for noninhalational HCN toxicity (eg, ingested cyanide, industrial or other occupational exposures); (2) reported hydroxocobalamin administration for conditions unrelated to HCN toxicity; (3) were review articles, meta-analyses, editorials, or conference abstracts without primary data; or (4) contained insufficient information to describe hydroxocobalamin use (eg, missing treatment setting or dose).

### 2.3 Data Collection and Outcomes of Interest

From each eligible study, data were extracted on study design, number of patients treated with hydroxocobalamin, patient demographic information, relevant predose laboratory data, hydroxocobalamin dose administered, time to administration, posttreatment outcomes (including survival), and AEs reported.

### 2.4 Data Synthesis and Analysis

All findings were summarized descriptively. Continuous variables are presented as means with standard deviations or medians with interquartile ranges, as reported. Categorical variables are summarized as counts and percentages. No inferential statistics or meta-analysis was performed, consistent with the objectives of a scoping review.

## 3 Results

### 3.1 Study Types and Geographic Locations

**Figure 1:**
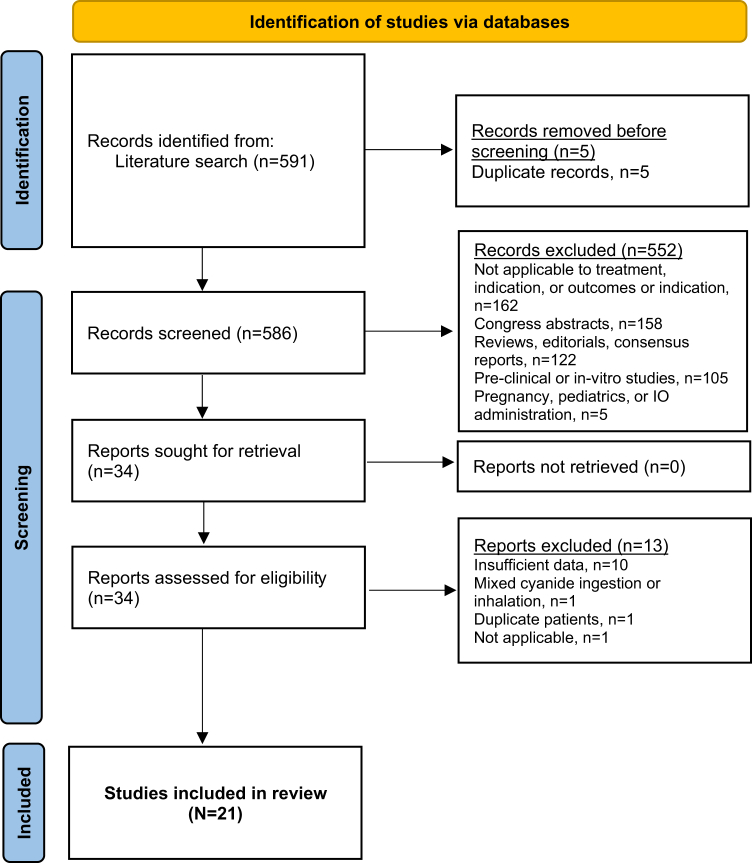
Flow diagram. IO, intraosseous.

After removing duplicates, 586 titles and abstracts were screened for eligibility. Of these, 34 full-text manuscripts were reviewed, and 21 met the inclusion criteria (Fig 1). Data extracted from each study are summarized in Table 1.

**Table 1:** Data extraction process

1. **Literature search** a. Request literature search in BIOSIS Previews, Embase, MEDLINE, PubMed, and Northern Light Life Sciences Conference Abstracts databases using search terms: ((cyanide or hydroxocobalamin) AND (inhalation injury or smoke inhalation)) b. Query authors for additional papers and/or abstracts c. Search the bibliographies of relevant papers for nonpeer-reviewed literature d. Identify duplicate records and remove them before review e. Obtain translation of abstracts if needed
2. **Eligibility evaluation** a. Review titles and abstracts for exclusion based on the following reasons: i. Type of publication (congress abstracts, reviews, editorials, and meta-analyses) ii. Study reporting preclinical research iii. Publication evaluating hydroxocobalamin use for another indication or type of cyanide exposure (not via smoke inhalation) iv. Publication not applicable to hydroxocobalamin treatment and/or evaluating nonadult population v. Publication containing insufficient data b. The remaining publications were retrieved and reviewed by the expert clinicians i. Additional exclusions were made if needed
3. **Data extraction** a. Type of study b. Number of patients treated and geographic region c. Demographic information d. Location of hydroxocobalamin administration (prehospital or in-hospital) e. Prehydroxocobalamin clinical data available f. Posthydroxocobalamin outcomes (including survival) g. Safety data reported
4. **Analysis**

The included studies comprised 13 case reports or series, 6 retrospective analyses, and 2 prospective analyses. Nine were conducted in the US,^20^, ^21^, ^22^, ^23^, ^24^, ^25^, ^26^, ^27^, ^28^ while 12 originated from the European Union (EU) or United Kingdom (UK)^29^, ^30^, ^31^, ^32^, ^33^, ^34^, ^35^, ^36^, ^37^, ^38^, ^39^, ^40^ (Table 2). Sample sizes ranged from single-patient reports to multicenter cohorts, reflecting substantial heterogeneity in study design and reporting quality.

**Table 2:** Overview of studies (N = 21)

	Citation	Study type	Timeframe studied (if applicable)	Geographic region
1	Baud 2011^29^	Case report	n/a	France
2	Borron 2007^30^	Prospective observational case series	6+ y	France
3	Cumpston 2021^20^	Retrospective, cross-sectional observational analysis	6 y	US
4	Datar 2019^21^	Case report	n/a	US
5	Davies 2020^31^	Case report	n/a	UK
6	Evans 2021^22^	Case report	n/a	US
7	Fortin 2006^32^	Retrospective chart review	8 y	France
8	Fortin 2007^34^	Case report	n/a	France
9	Fortin 2011^33^	Case report	n/a	France
10	Hamdini 2023^35^	Case report	n/a	France
11	Houeto 1995^36^	Prospective analysis	unk	France
12	Jiwani 2018^23^	Case report	n/a	US
13	Kamta 2023^24^	Retrospective observational analysis	10+ y	US
14	Kennedy 2020^37^	Case report	n/a	Ireland
15	Kiernan 2020^25^	Case report	n/a	US
16	Nguyen 2017^26^	Retrospective chart review	6 y	US
17	Pérez-Ajami 2024^38^	Case report	n/a	Spain
18	Pruskowski 2020^27^	Retrospective chart review	2+ years	US
19	Raska 2021^39^	Case report	n/a	Czech Republic
20	Torremadé Barreda 2010^40^	Case report	n/a	Spain
21	Wong 2022^28^	Retrospective chart review	11 y	US

n/a, not applicable; UK, United Kingdom; US, United States.

### 3.2 Patient Characteristics

Across the 21 studies, a total of 512 patients received hydroxocobalamin for suspected or confirmed HCN toxicity secondary to smoke inhalation (Table 3). Overall, 288 (56.3%) were male, and the patients ranged from 2 to 94 years old. Three studies also included pediatric patients.^24^^,^^28^^,^^32^ Clinical presentations commonly included altered mental status, loss of consciousness, or the presence of soot around the nose, mouth, or oropharynx. Many patients were hemodynamically unstable, apneic, pulseless, and/or in cardiorespiratory arrest upon presentation. Pretreatment laboratory data were inconsistently reported. Six studies provided HCN concentrations (range, 0-135 μmol/L), and 12 studies included serum lactate levels before hydroxocobalamin administration (range, 0.36 to >20 mmol/L).

**Table 3:** Treatment overview and clinical data of studies reporting intravenous (IV) hydroxocobalamin for management of smoke inhalation injury (N = 21)

	Citation	N	Male, n (%)	Age (y)^a^	Hydroxo-cobalamin administered: prehospital, hospital, or both	Total dose administered	Pretreatment data reported				Posttreatment outcomes reported		
							CO levels or Carboxy-Hgb	HCN	Lactate	Acid-base (ABG, VBG, pH, or AG)^b^	Lactate	LOS	Survival status
1	Baud 2011^29^	1	0 (0)	>50	Prehospital	2.5 g	√	√	---	---	√	√	√
2	Borron 2007^30^	69	33 (47.8)	44 (range, 20-94)	Prehospital	5-15 g	√	√	√^c^	---	√	---	√
3	Cumpston 2021^20^	42	23 (54.8)	46 ± 19	Prehospital	---	---	---	---	---	√	√	√
4	Datar 2019^21^	1	1 (100)	73	Both^d^	---	√	---	---	---	√	---	√
5	Davies 2020^31^	1	1 (100)	“middle-aged”	Hospital	5 g	√	---	√	√	√	√	√
6	Evans 2021^22^	1	1 (100)	54	Prehospital	---	---	---	---	---	√	---	√
7	Fortin 2006^32^	101^e^	53 (52.5)	48.5 (range, 2-88)	Prehospital	Median 5 g (1-10)	---	---	---	---	---	---	√^f^
8	Fortin 2007^34^	1	0 (0.0)	47	Prehospital	10 g	---	---	---	---	---	√	√
9	Fortin 2011^33^	1	1 (100)	23	Both^d^	10 g	---	√	√	---	---	√	√
10	Hamdini 2023^35^	1	0 (0.0)	76	Prehospital	10 g	---	---	---	---	√	√	√
11	Houeto 1995^36^	12	6 (50)	56.5 ± 25.2	Both^g^	5 or 10 g^h^	√	√	---	---	---	---	√^i^
12	Jiwani 2018^23^	1	1 (100)	47	Hospital	5 g	√	---	√	√	√	---	---
13	Kamta 2023^24^	46^e^	30 (65.2)	44 ± 23	Both^j^	5 or 10 g^j^	√	---	√	√	√	√	√
14	Kennedy 2020^37^	1	1 (100)	78	Hospital	---	---	---	√	√	√	---	√
15	Kiernan 2020^25^	1	1 (100)	62	Hospital	5 g	√	√	√	√	---	√	√
16	Nguyen 2017^26^	138	71 (51.4)	54 (IQR 40-65)	Hospital	---	√	---	√	√	---	√	√
17	Pérez-Ajami 2024^38^	1	1 (100)	44	Both^d^	15 g	---	---	---	---	√	√	√
18	Pruskowski 2020^27^	35	23 (65.7)	46 (IQR 34.5-65.8)	Hospital	5 or 10 g^k^	√^l^	---	√	---	√	√	√
19	Raska 2021^39^	2	1 (50.0)	53.5 ± 10.6	Hospital	5 g	---	---	√^m^	√	√	---	√
20	Torremadé Barreda 2010^40^	1	1 (100)	59	Prehospital	5 g	√	---	√	---	---	---	√
21	Wong 2022^28^	55^n^	39 (70.9)	52 (IQR 42-62)	Hospital	5 g^o^	√	√^p^	√	√	---	√	√

ABG, arterial blood gas; AG, anion gap; CO, carbon monoxide; HCN, hydrogen cyanide; LOS, length of stay; VBG, venous blood gas.

a Age reported as described in the studies: mean ± SD or median (range or interquartile range [IQR]).

b Acid-base status reported as an ABG, VBG, just pH or anion gap.

c Pretreatment lactate obtained in some patients.

d Patient received 1 dose before hospitalization and additional dose(s) in the emergency department.

e Unclear how many were pediatric patients.

f Survival status was not known/reported for 29 patients.

g Seven patients received hydroxocobalamin prior to hospitalization and 5 received their dose in the hospital.

h 10 patients received 5-g dose, 2 patients received 10 g total.

i Survival status was reported for all 12 patients, but not by location of hydroxocobalamin administration.

j Eight patients received doses in the prehospital setting and 38 received hydroxocobalamin while hospitalized; 43 patients received “1 dose” and 3 patients received “a second dose”, presumably 5 and 10 g, respectively.

k 31 patients received “1 dose” and 4 patients received “2 doses”, presumably 5 and 10 g, respectively.

l Carboxyhemoglobin levels reported as >10% in 1 patient and < 3% in 4 patients.

m For one patient, pretreatment lactate was reported.

n N = 55 includes 4 children, but age reported is only for adults.

o All adults received a 5-g dose.

p HCN levels reported in 17 cases.

### 3.3 Hydroxocobalamin Use

Thirteen studies described prehospital administration of hydroxocobalamin, representing 235 out of the 512 (45.9%) patients. Reported total doses of hydroxocobalamin were most commonly 5 or 10 g, although 5 studies did not specify the amount administered. Time to hydroxocobalamin administration was reported in 5 studies^23^, ^24^, ^25^^,^^28^^,^^32^ and ranged from a mean of 14 minutes among 101 patients to 19 hours in 1 patient.

Additional therapies were occasionally used. Hyperbaric oxygen therapy was reported in 3 studies (n = 59 patients) for concomitant carbon monoxide (CO) poisoning. Sodium thiosulfate was co-administered or administered sequentially in 4 patients;^21^^,^^24^^,^^31^^,^^39^ and dicobalt edetate was used in 1 case before hydroxocobalamin.^31^

### 3.4 Clinical Outcomes

Posttreatment outcomes varied among studies (Table 3). Improvements in neurologic status, lactate clearance, acid-base balance, time on ventilator, and hospital length of stay were reported. Across 20 studies with survival data (n = 482 patients), 318 (66.0%) survived to hospital discharge. When stratified by treatment location, survival was 62.3% (124/199) for patients who received hydroxocobalamin in the prehospital setting and 69.0% (187/271) for those treated at the hospital. One study reported that the median time to administration in survivors was 36 minutes quicker than in those who died (185 minutes vs 221 minutes).^28^

When limited to noncase report studies (n = 498), the overall survival rate was similar at 66.3% (311 of 469 patients with known survival status). Among these, 61.3% (117/191) of prehospital-treated and 69.5% (185/266) of hospital-treated patients survived to hospital discharge.

### 3.5 Adverse Events

AEs were described in 13 studies encompassing 69 patients. Common and expected AEs were chromaturia (15/512, 2.9%)^21^^,^^22^^,^^30^^,^^32^^,^^37^^,^^40^ and skin discoloration/erythema/rash (9/512, 1.8%).^21^^,^^29^^,^^30^^,^^32^

Elevated blood pressure was reported in 5 patients.^30^ Acute kidney injury (AKI) occurred in 38 patients across 5 studies.^22^^,^^24^^,^^27^^,^^35^^,^^38^ Of these, 2 were attributed to oxalate nephropathy.^22^^,^^35^ AKI resolved in most patients (33/38, 86.8%) prior to discharge; 5 (13.2%) patients required continuation of renal replacement therapy after hospital discharge. Methemoglobinemia was reported in 2 (0.4%) patients.^23^^,^^25^

## 4 Limitations

This scoping review is subject to several important limitations. First, the evidence base consists predominantly of observational studies, case series, and case reports, with no randomized or controlled trials identified. Consequently, study designs, patient populations, and clinical settings varied widely, resulting in heterogeneity in dosing, timing, and outcome reporting. The lack of comparative investigations precludes assessment of causal relationships or direct evaluation of hydroxocobalamin efficacy relative to other interventions.

Second, the completeness of the data was inconsistent across studies. Key variables, such as time from exposure to antidote administration, concurrent carbon monoxide levels, and pretreatment and posttreatment laboratory measures, were often missing or incompletely reported. Although earlier administration is presumed to be most beneficial, the limited and inconsistent reporting of timing prevented meaningful analysis of its impact on survival or neurologic outcomes.

Third, while this review focused on adult patients with smoke inhalation-related cyanide toxicity, 2 of the included studies contained small numbers of pediatric patients.^28^^,^^32^ Dedicated pediatric studies, as well as reports involving pregnancy or alternate routes of administration (eg, intraosseous), were excluded; therefore, these results should not be extrapolated to those populations.

Finally, as a descriptive scoping review, this analysis was not intended to assess treatment efficacy but rather to map the existing evidence, identify gaps, and characterize real-world practice patterns. Despite these limitations, this synthesis highlights critical areas for future research and opportunities to standardize data collection and optimize hydroxocobalamin use in both prehospital and hospital settings.

## 5 Discussion

This scoping review identified 21 studies describing hydroxocobalamin use for management of HCN toxicity secondary to smoke inhalation in a closed-space fire. Most investigations were small, observational, or case-based, reflecting the limited formal study of this life-threatening but infrequent condition. Substantial heterogeneity was observed in the study design, patient selection, dosing strategies, and pretreatment and posttreatment assessments. Collectively, these findings underscore wide variability in the recognition, management, and reporting of suspected HCN toxicity following closed-space fires.

### 4.1 Patterns of Use and Geographic Variation

Of the included studies, nearly half described prehospital hydroxocobalamin administration, though most were case reports, and few occurred within the US. Only 4 of 9 US-based studies reported prehospital use (n = 52) compared with 9 of 12 UK- or EU-based studies (n = 183). This finding highlights potential regional differences in training, antidote availability, and prehospital treatment protocols.

Among the studies reporting survival outcomes, survival was modestly lower in patients treated prehospital compared with those in the hospital. This difference likely reflects confounding by illness severity, as those receiving field antidotes may have presented in extremis. Although hydroxocobalamin’s rapid mechanism of action supports early administration, few studies documented time to treatment, precluding evaluation of its effect on outcomes. Given cyanide’s short half-life (1-3 hours),^41^ the potential benefits of early administration warrant further investigation, particularly in Emergency Medical Services (EMS) systems capable of providing the antidote at the scene.

### 4.2 Safety and AEs

AEs were infrequently reported and consistent with the known safety profile of hydroxocobalamin. Common and expected effects, such as skin discoloration, chromaturia, and mild elevations in blood pressure, are generally transient and clinically benign.^42^ Notably, transient blood pressure elevation may be advantageous in cyanide-induced cardiovascular collapse. AKI occurred in approximately 7% of treated patients, with 2 cases attributed to oxalate nephropathy. However, most patients experiencing AKI were critically ill with burn or inhalation injury, and renal dysfunction likely reflected multifactorial causes, such as hemodynamic instability, systemic inflammation, and sepsis, among other factors.^43^ In nearly 90% of cases, renal function recovered before hospital discharge. Fluid resuscitation is an imperative part of managing patients with burns,^43^^,^^44^ and continuous monitoring of renal function for several days remains prudent (Table 4).^17^

**Table 4:** Overview of Hydroxocobalamin^17^

Mechanism of action	Hydroxocobalamin binds cyanide ions, forms cyanocobalamin, and is then excreted in the urine
FDA-approved indication	Treatment of known or suspected cyanide poisoning
Dosing	For adults: 5 gm IV over 15 minutes A second dose (5 gm) may be administered if needed based on the severity of the poisoning and clinical response (administered over 15 minutes – 2 hours)
Common AEs	Transient chromaturia, erythema, oxalate crystals in urine, rash, increased blood pressure, nausea, headache, infusion site reactions
Warnings and precautions	Monitor for: • Anaphylactic or other hypersensitivity reactions • Acute renal failure with acute tubular necrosis, renal impairment, and urine calcium oxalate crystals • Elevations in blood pressure • Interference with colorimetric interpretation of certain laboratory tests due to the deep red color

### 4.3 Barriers to Antidote Use

Despite more than 2 decades since US FDA approval, hydroxocobalamin use remains inconsistent across regions and agencies. Barriers include limited awareness, lack of training, absence of standardized protocols, and cost considerations. Surveys of fire and EMS departments reveal that fewer than one-fifth maintain formal treatment protocols for cyanide toxicity in smoke inhalation victims, despite frequent smoke-exposure incidents among firefighters.^19^

**Figure 2:**
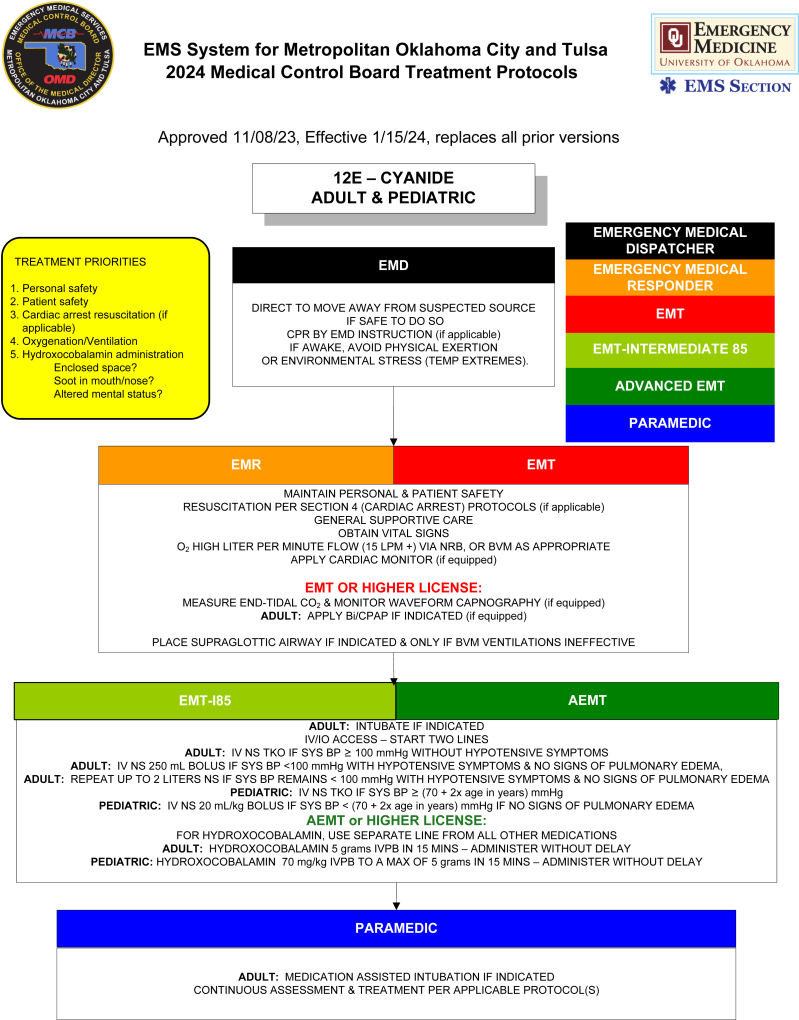

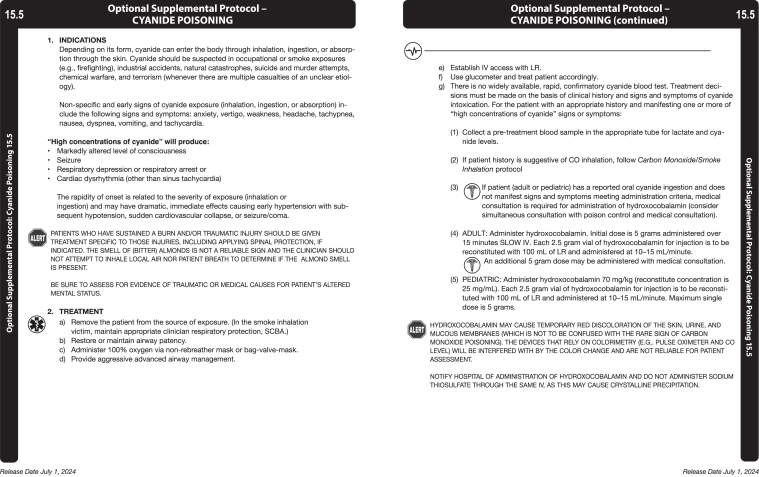
Sample prehospital treatment protocol for management of hydrogen cyanide (HCN) poisoning and use of hydroxocobalamin in the setting of smoke inhalation.^a^ (A) Oklahoma City and Tulsa.^47^ (B) Maryland.^48^^a^This is meant to serve as a reference or framework for development of a protocol for use by EMS or fire rescue agency; other protocols may also be applicable. Used with permission from the Oklahoma Medical Control Board and Maryland Institute for Emergency Medical Services Systems.

The National Institute for Occupational Safety and Health (NIOSH) has emphasized the need to prevent on-duty deaths from cardiovascular causes among firefighters^45^^,^^46^ and to mitigate exposure to toxic gases such as CO and HCN.^8^ Expanding education and providing clear guidance for EMS and fire personnel regarding antidote indications, dosing, and AE management could enhance timely and appropriate use.^20^ Sample protocols from the Oklahoma City and Tulsa EMS System (Fig 2A)^47^ and the Maryland Institute for Emergency Medical Services Systems (Fig 2B)^48^ illustrate frameworks that could be adopted more broadly to improve readiness.

Cost remains a persistent barrier. Each 5-g vial costs roughly $1000 [data on file, SERB Pharmaceuticals (previously BTG)] and has a limited shelf-life, necessitating periodic replacement. However, modeling studies suggest that early antidote use may reduce downstream healthcare costs through shorter hospital stays and less prolonged mechanical ventilation.^26^^,^^49^ Regional approaches to purchasing, state-level stockpiles, or grant-based funding may help mitigate these financial constraints.

### 4.4 Implications and Future Directions

The available evidence supports hydroxocobalamin as a well-tolerated, mechanistically sound antidote for suspected HCN poisoning from smoke inhalation. Nevertheless, the fragmented and largely descriptive nature of existing data limits definitive conclusions regarding optimal timing, dosing, or patient selection. Future studies should prioritize standardized data collection, including clearly defined exposure metrics, and examine outcomes based on time to administration and care setting. Development of uniform prehospital protocols, supported by education and cost-sharing strategies, could improve access and reduce treatment delays.

In summary, current literature describing hydroxocobalamin use for HCN toxicity caused by smoke inhalation from fires is limited but suggests favorable outcomes and a low AE profile consistent with AHA recommendations. The observed variability in practice reflects broader gaps in recognition, access, and system preparedness for managing HCN toxicity. Hydroxocobalamin remains the preferred antidote for suspected cyanide poisoning and is particularly suitable for prehospital use. Expanding training, protocol adoption, and equitable access to this therapy represent key opportunities to improve survival following closed-space fire exposure.

## Author Contributions

RD: conceptualization, methodology, project administration, supervision, writing-review, and editing. JMG, JJA, EC: writing-review and editing. RPC: conceptualization, visualization, writing review, and editing. TB: conceptualization, funding acquisition, methodology, writing-review, and editing.

## Funding and Support

Medical writing and editorial assistance were funded by 10.13039/100014869BTG International Inc.

## Conflict of Interest

TB is an employee of BTG International Inc.
